# Editorial: Muscle dysfunction of critical illness

**DOI:** 10.3389/fphys.2023.1336150

**Published:** 2023-11-27

**Authors:** Dmitry Rozenberg, Sunita Mathur, Jane Batt

**Affiliations:** ^1^ Toronto General Hospital Research Institute, Ajmera Transplant Center, University Health Network, Toronto, ON, Canada; ^2^ Division of Respirology, Department of Medicine, Temerty Faculty of Medicine, University of Toronto, Toronto, ON, Canada; ^3^ School of Rehabilitation Therapy, Queen’s University, Kingston, ON, Canada; ^4^ Keenan Research Center for Biomedical Science, Unity Health, Toronto, ON, Canada

**Keywords:** skeletal muscle, muscle wasting, function, ultrasound, ICU-acquired weakness, microcirculation

Intensive care unit acquired weakness (ICUAW) is a common complication of critical illness and associated with increased morbidity and mortality (Herridge and Azoulay, 2023). Both respiratory muscle weakness and limb muscle dysfunction occur with critical illness, which can affect ICU and hospital length of stay, weaning from mechanical ventilation (MV) and functional recovery (Vanhorebeek et al., 2020). This thematic Research Topic of five articles highlights the complex interplay between skeletal muscle mass, strength and function in the critical care setting. The evaluation of muscle quality with ultrasound is highlighted and associations with fiber size and collagen deposition in the extra-cellular matrix are demonstrated. Muscle cell cytoarchitecture and microvascular dysfunction are discussed.

The article by Chueire de Andrade-Junior et al. characterizes skeletal muscle mass and function in 32 patients with severe COVID-19 infection admitted to the critical care unit. The investigators observed a significant decline in quadriceps muscle size with ultrasound (ranging from 19% to 30%) and decreased hand-grip strength (22%) within 10 days of ICU admission. However, there was recovery demonstrated by day 10 of ICU care with the ICU mobility scale and respiratory structure and function assessed with the International Classification of Functioning Disability and Health Assessment. Similar findings were observed by Silva-Gutierrez et al. who evaluated 132 patients in the ICU setting on MV due to severe COVID-19 infection. Quadriceps muscle size (vastus intermedius, rectus femoris) decreased by 10%–20%, whereas muscle strength and mobility improved on ICU discharge. Risk factors of older age (≥60 years old) and longer MV course (>10 days) were associated with greater decline in muscle mass and lower strength and functional mobility. These two articles highlight important known risk factors for ICUAW shown in other critical care populations, specifically older age, severity of illness, and prolonged ICU stay (Puthucheary et al., 2013; Lad et al., 2020; Vanhorebeek et al., 2020).

Mayer et al. extends the findings in critical care survivors demonstrating that better muscle quality, measured using echogenicity from ultrasound images was associated with greater limb muscle strength, physical function, muscle fiber size and fewer alterations in collagen at the extracellular matrix. The findings from Mayer et al. reinforce that echogenicity from ultrasound can be utilized as a measure of muscle quality in the critical care setting and may be a surrogate measure of muscle function (Puthucheary et al., 2015). The investigators highlight that these relationships between muscle quality and function are similar in a diverse group of study participants recovering from COVID-19 infection (mild and severe COVID infection) and healthy participants. This study provides further support for the validity of utilizing ultrasound as an assessment of skeletal muscle when biopsies are not readily available or feasible to conduct.

Diaphragm weakness also occurs in the MV critically ill patient due to atrophy and impaired contractility, and is referred to as ventilator induced diaphragm dysfunction (VIDD) (Spiesshoefer et al., 2023). The diaphragm is unique however, in that weakness occurs more rapidly than in peripheral skeletal muscle, and myofiber specific force has been reported to fall prior to the development of single myofiber atrophy. While bioenergetics failure can contribute to impaired muscle contractility, structural integrity of the myofibrillar lattice in single fibres is a major determinant of integrated force production of the multi-myofibrillar array and has not been assessed in diaphragms of either critically ill humans or animal models of VIDD. In their study, Mnuskina et al. use label-free multiphoton Second Harmonic Generation (SHG) imaging followed by quantitative morphometry in single diaphragm muscle fibres from healthy rats subjected to MV + neuromuscular blockade. This enabled the assessment of the relative orientation and registry of adjacent myofibrils along the fibre length, to determine angular deviations in 3D and metrics of lattice organization as predictors of structure-related muscle weakness. They demonstrate that myofibrillar disarray occurs within 5 days of MV, well to diaphragm myofiber atrophy. They also demonstrate that treatment of the mechanically ventilated rats with BGP-15 or Vamorolone (VBP-15) improved diaphragm muscle specific force. BGP-15, a heat shock protein chaperone co-inducer known to inhibit post-translational myofibrillar protein modifications had no effect on fibre atrophy but significantly improved myofiber force by partially restoring the angular disarray of myofibrils in single fibres. VBP-15, a glucocorticoid that selectively activates anti-inflammatory signalling while minimizing activation of ‘pro-myopathy’ events, improved contractility by restoring axial lattice order. Using the novel approach of quantitative single fibre morphometry, the investigators demonstrate how altered cytoarchitecture contributes to decreased myofiber specific force and VIDD.

While changes within the myocyte and myofiber result in the loss of muscle mass and impaired contractility that characterize ICUAW and VIDD, the review by Mendelson and colleagues highlights the importance of a well-regulated microvascular system for normal muscle function and strength. Dysregulation of the microvascular circulation is now recognized to be a central player in the pathogenesis of critical illness (Ostergaard et al., 2015), and while a definitive causative role in ICUAW or VIDD remains to be determined, research is emerging supporting this notion. For example, disruption in microvascular bulk flow in sepsis impairs insulin delivery to skeletal muscle and glucose homeostasis, and hyperglycemia is a risk factor for ICUAW (Yang et al., 2018). Tissue hypoxia and release of inflammatory mediators from damaged endothelium can induce muscle proteolysis and impair satellite cell activation, resulting in muscle wasting and impaired regenerative/repair capacity (Friedrich et al., 2015; Lad et al., 2020). Moreover, the authors propose that ICU patients experience physiological defects along the complete oxygen delivery pathway–from “mouth to mitochondria”, restricting oxygen consumption and culminating in muscle weakness and impaired exercise capacity. A better understanding of the role of skeletal muscle microcirculation and microvascular milieu in initiating, propagating, and sustaining ICUAW is essential as it may provide novel opportunities for treatment, beyond the current focus of direct manipulation of the myocyte itself.

This Research Topic highlights the complexity of the pathophysiology underpinning ICUAW, and the interplay between muscle size, quality and function. The mechanisms both intrinsic to the myocyte (atrophy, impaired structural integrity and contractility) and those extrinsic via the microcirculatory environment are emerging as important factors of skeletal muscle dysfunction with critical illness.
